# Supplementary material for: Quantum Magnetic Properties in Perovskite with Anderson Localized Artificial Spin‐1/2
Source: Adv Sci (Weinh). 2018 Mar 2;5(5):1700978. doi: 10.1002/advs.201700978 (PMC5980209; doi:10.1002/advs.201700978)
Supplement: Supplementary file 1 — Supplementary [file ADVS-5-1700978-s001.pdf]

Copyright WILEY-VCH Verlag GmbH & Co. KGaA, 69469 Weinheim, Germany, 2018.

ADVANCED SCIENCE
Open Access

Supporting Information

for *Adv. Sci.,* DOI: 10.1002/advs.201700978

Quantum Magnetic Properties in Perovskite with Anderson Localized Artificial Spin-1/2

*Jagath Gunasekera, Ashutosh Dahal, Yiyao Chen, Jose A. Rodriguez-Rivera, Leland W. Harriger, Stefan Thomas, Thomas W. Heitmann, Vitalii Dugaev, Arthur Ernst, and Deepak K. Singh**

Supporting Information: Quantum Magnetic Properties in Perovskite with Anderson Localized Artificial Spin-1/2

Jagath Gunasekera[1], Ashutosh Dahal[1], Yiyao Chen[1], Jose A. Rodriguez-Rivera[2,3], Leland W. Harriger[2], S. Thomas[4], Thomas W. Heitmann[5], Vitalii Dugaev[6], Arthur Ernst[4,7], and Deepak K. Singh[1,*]
[1]*Department of Physics and Astronomy, University of Missouri, Columbia, MO, USA*
[2]*NIST Center for Neutron Research, Gaithersburg, MD, USA*
[3]*Department of Materials Science and Engineering, University of Maryland, College Park, MD,USA*
[4]*Max-Planck-Institut für Mikrostrukturphysik, Weinberg 2, 06120 Halle, Germany*
[5]*University of Missouri Research Reactor, Columbia, MO, USA*
[6]*Department of Physics and Medical Engineering, Rzeszów University of Technology, Rzeszów, Poland*
[7]*Institut für Theoretische Physik, Johannes Kepler Universität, 4040 Linz, Austria and*
[*]*email: singhdk@missouri.edu*

SAMPLE PREPARATION AND X-RAY DIFFRACTION ANALYSIS OF SAMPLE QUALITY

The high purity polycrystalline samples of $Ca(Co_xRu_{1-x})O_3$ were synthesized by conventional solid state reaction method using ultra-pure ingredients of CoO, RuO_2 and $CaCO_3$. Starting materials were mixed in stoichiometric composition, pelletized and sintered at 950° for three days. The furnace-cooled samples were grinded, pelletized and sintered at 1000° for another three days. Samples were intentionally synthesized at slightly lower temperature and for longer duration to preserve the oxygen stoichiometry. Resulting samples were characterized using Siemens D500 powder X-ray diffractometer, confirming the single phase of material. The X-ray diffraction data were analyzed using the FullProf suite for the Reitveld refinement, confirming the high quality single phase of materials (see Figure S1). The refined parameters, such as Wyckoff positions, atomic positions and lattice parameters for various substitution coefficients x are tabulated below in Table 1.

ELECTRICAL AND MAGNETIC MEASUREMENTS OF THE SYNTHESIZED SAMPLES

Four probe technique was employed to measure electrical properties of $Ca(Co_xRu_{1-x})O_3$ using a closed-cycle refrigerator cooled 9 T magnet with measurement temperature range of 1.5-300 K. Detailed ac susceptibility measurements were performed using a Quantum Design Physical Properties Measurement System with a temperature range of 2-300 K.[1]

FITTING OF STATIC SUSCEPTIBILITY USING MEAN-FIELD CALCULATION

We have performed preliminary mean-field calculations to estimate the strength of Co-Co and Ru-Ru exchange interactions. In the following calculation, J_{Co-Co} represents the Co-Co interaction and J_{Ru-Ru} represents Ru-Ru interaction.

In the mean field approximation, the magnetization density is written as,[2]

$$M = M_0 \frac{H_{eff}}{T}, \tag{1}$$

where $H_{eff} = H + \lambda M$. λ is related to J via following relation,

$$J_i = \frac{\lambda_i}{k_B}(g\mu_B)^2, \tag{2}$$

where (i = 1,2). In the case of two coupled sub-lattices, the above equation is modified to the following general form, [2]

$$M_1 = \frac{M_0[H + \lambda_1 M_1 + \lambda_3 M_2]}{T}, \tag{3}$$

$$M_2 = \frac{M_0[H + \lambda_2 M_2 + \lambda_3 M_1]}{T}, \tag{4}$$

For simplicity, we write $J_1 = J_{Co-Co}$ and $J_2 = J_{Ru-Ru}$. As mentioned in Ref. [2], above equations can be further simplified to,

$$H_1 = 1 + \lambda_1\chi_1 + \lambda_3\chi_2, \tag{5}$$

$$H_2 = 1 + \lambda_2\chi_2 + \lambda_3\chi_1, \tag{6}$$

where one can write χ(T) for a $3d$-ion as

$$\chi_i = \frac{\chi_i(0)^2 S_i(S_i+1)H_i}{T}. \tag{7}$$

For two magnetic ions, the bulk or total susceptibility will be given by,

$$\chi(T) = \chi_1(T) + \chi_2(T), \tag{8}$$

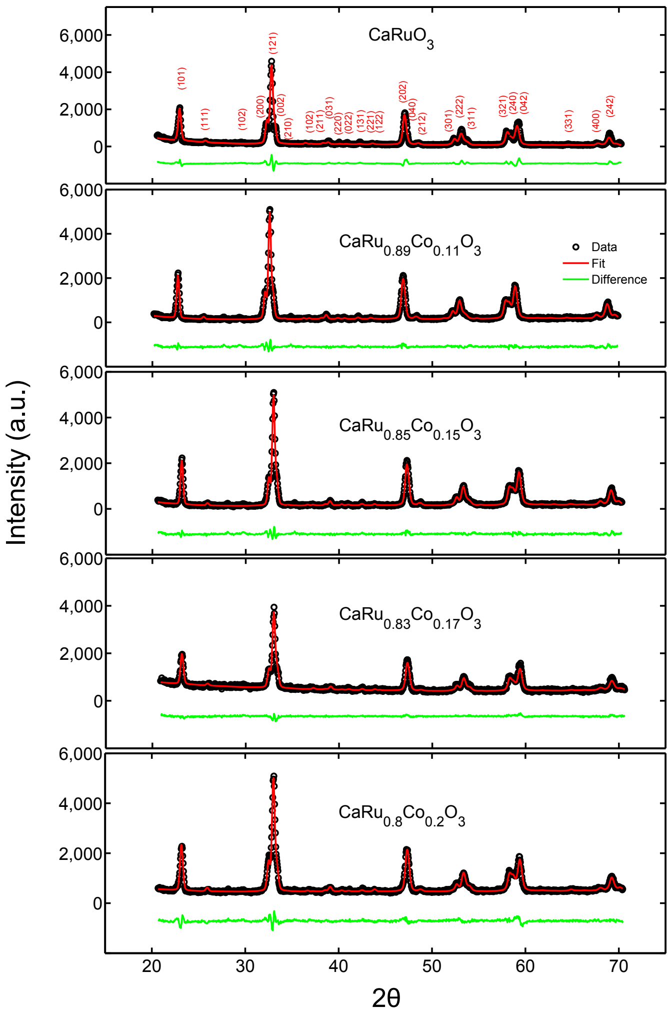

FIG. S1: **XRD profiles of $Ca(Co_xRu_{1-x})O_3$.** Powder X-ray diffraction spectra of $Ca(Co_xRu_{1-x})O_3$ powder, for various substitution coefficient x, used in this study. The powder diffraction data is refined using FullProf suite for the Rietveld analysis. The high purity of the sample is evident from the XRD data, where every single peak is identified with the orthorhombic structure of $CaRuO_3$.

$$\chi(T) = \frac{(C_1+C_2)T - C_1C_2(\lambda_1+\lambda_2-2\lambda_3)}{(T-C_1\lambda_1)(T-C_2\lambda_2)-C_1C_2\lambda_3}, \quad (9)$$

where $C_1 = \chi_1^2(0).S_1(S_1{+}1)$ and $C_2 = \chi_2^2(0).S_2(S_2{+}1)$.

We have fitted the susceptibility data of x = 0.19 composition in Figure 2d using the above equation to obtain λ_i that in turn are related to J_{Co-Co} and J_{Ru-Ru}. The fitted values of J_{Co-Co} and J_{Ru-Ru} are found to be -5.6 meV and 0.19 meV, respectively.

HEAT CAPACITY MEASUREMENTS

We have performed heat capacity measurements on the substitution coefficient x = 0.17 sample in applied fields of H = 0 and 5 T utilizing the QD PPMS with a base temperature of T = 1.8 K. The background corrected

TABLE I: Wyckoff positions, atomic positions and lattice parameters for various substitution coefficients x, as obtained from the refinement of the XRD spectra using FullProf suite, are presented here.

$CaRuO_3$	Wyck	x	y	z
Ca	4c	0.9536	0.25	0.015
Ru	4b	0	0	0.5
O(1)	8d	0.192	0.446	0.215
O(2)	4c	0.022	0.25	0.5899

$CaRu_{0.89}Co_{0.19}O_3$	Wyck	x	y	z
Ca	4c	0.954	0.25	0.006
Ru,Co	4b	0	0	0.5
O(1)	8d	0.196	0.446	0.214
O(2)	4c	0.031	0.25	0.586

$CaRu_{0.85}Co_{0.15}O_3$	Wyck	x	y	z
Ca	4c	0.955	0.25	0.005
Ru,Co	4b	0	0	0.5
O(1)	8d	0.198	0.446	0.216
O(2)	4c	0.0307	0.25	0.584

$CaRu_{0.83}Co_{0.17}O_3$	Wyck	x	y	z
Ca	4c	0.956	0.25	0.0052
Ru,Co	4b	0	0	0.5
O(1)	8d	0.190	0.446	0.217
O(2)	4c	0.043	0.25	0.587

	a (Å)	b (Å)	c (Å)
$CaRuO_3$	5.545	7.673	5.398
$CaRu_{0.89}Co_{0.19}O_3$	5.537	7.665	5.387
$CaRu_{0.85}Co_{0.15}O_3$	5.533	7.664	5.378
$CaRu_{0.8}Co_{0.2}O_3$	5.514	7.662	5.37

data are shown in Figure S3. In the heat capacity plots, no sharp cusp indicating transition to a magnetic ordered state is observed. Also noticeable is the absence of magnetic field dependence of the heat capacity up to H = 5 T. Given the fact that the magnetic gap arises at E = 5.9 meV, which is equivalent to $H \simeq 40$ T for spin-1/2, it is no surprise that heat capacity is independent of field up to H = 5 T. Also, the lack of any upturn in the heat capacity data at low temperature suggest the absence of the nuclear Schottky-type effect or the cluster spin glass behavior.

NEUTRON SCATTERING EXPERIMENT

Elastic measurements: 2θ-scans were carried out on a thermal triple-axis spectrometer TRIAX at the Missouri University and on a cold triple axis spectrometer, SPINS, at the NIST Center for Neutron Research. Measurements on the thermal spectrometer was performed

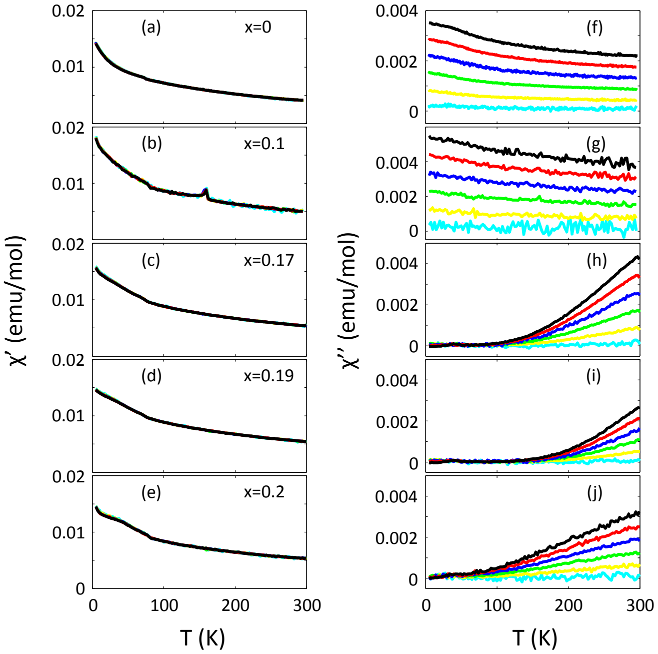

FIG. S2: **AC susceptibilities of Ca(Co$_x$Ru$_{1-x}$)O$_3$.** The static and dynamic susceptibilities as a function of temperature at different frequencies between 500 - 10^4 Hz, as obtained from the ac susceptibility measurements, for many substitution coefficients. While the static susceptibilities are ac frequency independent, the dynamic susceptibilities exhibit strong frequency dependences. Most notably, the $\chi^{"}$ exhibits a complete reversal in the fluctuation spectrum as a function of frequency across the critical doping x = 0.15. $\chi^{"}$ is much stronger at higher temperatures in x > 0.15.

with a fixed final energy of 14.7 meV and the collimation setting of 60'-PG filter -monochromator -PG (pyrolitic graphite) filter-80'-sample-40'-PG filter -PG analyzer-80'-detector. Measurements on SPINS were performed at the fixed final energy of E_f =5 meV with the collimators sequence of PG (mono)-Be Filter-Sample-BeO filter-80'-flat analyzer-Detector. As shown in Figure S4, no evidence of magnetic order is found in compounds with x > 0.15.

Inelastic measurements: The temperature and magnetic field dependences of the dynamic properties were investigated in various compositions of Ca(Co$_x$Ru$_{1-x}$)O$_3$ using inelastic neutron scattering measurements on the SPINS cold triple-axis spectrometer and MACS spectrometer at the NIST Center for Neutron Research. The spectrometer's configuration for inelastic measurements on SPINS was PG (mono)-80'-Sample-BeO filter-radial collimator-10 blades focused analyzer-Detector. The spectrometer's resolution was determined using an empty vanadium can scan. At $E_f = 5$ meV, the spectrometer's resolution was determined to be $\simeq 0.28$ meV. The spectrometer's configuration on MACS was PG (mono)-80'-Sample-Be filter-radial collimator- focused analyzer- 20 detectors. Measurements were performed for the fixed

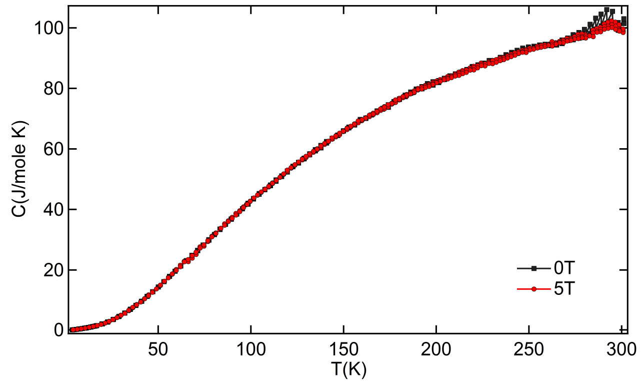

FIG. S3: **Heat capacity data of Ca(Co$_{0.17}$Ru$_{0.83}$)O$_3$.** Heat capacity measurements performed on substitution coefficient x = 0.17 in applied magnetic field using a QD PPMS with a base temperature of $T = 1.8$ K. No signature of a transition to the ordered magnetic state is observed in the heat capacity data.

final energy of $E_f = 5$ meV, at which the spectrometer's resolution was determined to be $\simeq 0.32$ meV. In Figure S5, we plot the raw data at two characteristic measurement temperatures of $T = 30$ K and 80 K at two magnetic fields of $H = 0$ T and $H = 9$ T, the background data and the background corrected data. Figure S6 depicts measurements on SPINS in both zero and applied field of H = 10 T at two characteristic temperatures of $T = 5$ K and 100 K.

NORMALIZATION OF INELASTIC NEUTRON SCATTERING DATA USING A VANADIUM SAMPLE OF KNOWN MASS

Inelastic data were also normalized with respect to the incoherent scattering from a vanadium sample of known mass. Using the method mentioned in Ref. [3], we write S(Q, ω) as,[3]

$$S(Q,\omega) = \frac{13.77(barn)^{-1}{\mu_B}^2 I(Q,E)}{F(Q)^2 e^{-2W} N k_f R_0}. \qquad (10)$$

The energy integrated incoherent scattering intensity,

$$\int I(Q,E)\,dE = \frac{N}{4\pi}\sum_j {\sigma_j}^{inc} e^{-2W} k_f R_0 \qquad (11)$$

was obtained by performing $\Delta E = 0$ scan at a wave vector Q. Therefore, we have

$$N k_f R_0 = 4\pi \frac{\int I(Q,E)\,dE}{\sum_j {\sigma_j}^{inc} e^{-2W}}. \qquad (12)$$

In the calculations we have neglected the variation of Debye-Waller factor ($e^{-2W} \simeq 1$). The integrated intensities were obtained by fitting the data that have been

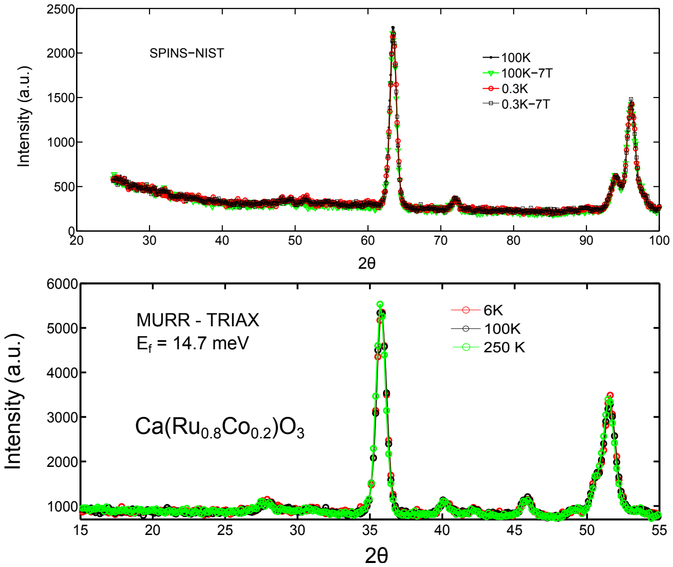

FIG. S4: **Elastic measurement of $Ca(Co_xRu_{1-x})O_3$.** Top panel- Elastic measurements performed on the substitution coefficient x = 0.17 on a cold spectrometer SPINS using high resolution collimation and filters, with fixed final energy of E_f = 5 meV, to a low temperature of T = 0.3 K and in applied field of H = 7 T. Elastic neutron scattering pattern at various temperatures and fields are presented in this figure. Clearly, no extra peak, beyond the lattice peaks, is detected. Also, no enhancement in the lattice peaks or a diffuse background associated to the elastic peak are observed at low temperature. Together, they rule out any magnetic order in the system. Bottom panel- Elastic measurements performed on a thermal triple axis spectrometer TRIAX (at MURR) on the substitution coefficient x = 0.2 further confirms the absence of magnetic order in $Ca(Co_xRu_{1-x})O_3$.

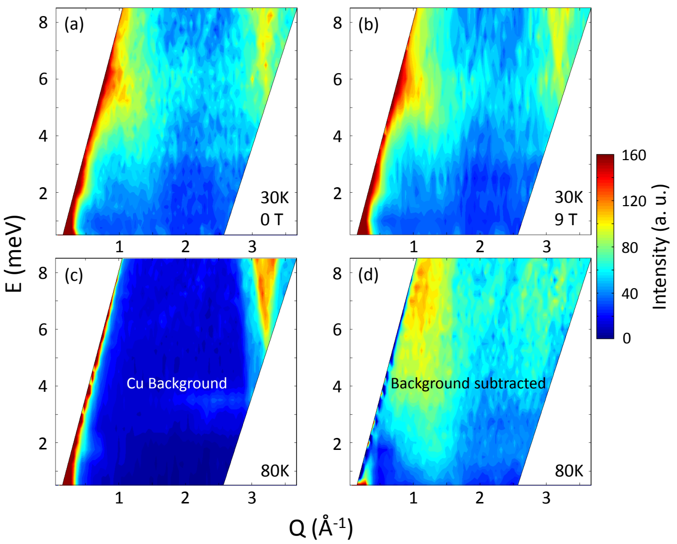

FIG. S5: **Color map of spin fluctuation in energy-momentum space, as obtained from the MACS measurements**. (a) and (b) represents raw data at T = 30 K at H = 0 T and 9 T, respectively. (c) Cu background data at T = 80 K. Sharp scattering at high Q is due to copper can. (d) Background corrected and thermally normalized data at T = 80 K.

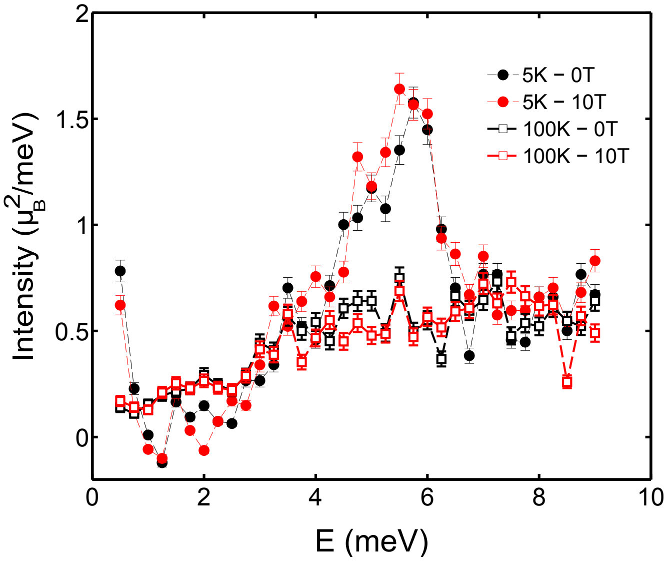

FIG. S6: **Background corrected inelastic data in both zero and applied field of H = 10 T at T = 5 K and 100 K**. Clearly, no field dependence is observed at any temperature. Measurements were performed on the SPINS cold triple axis spectrometer.

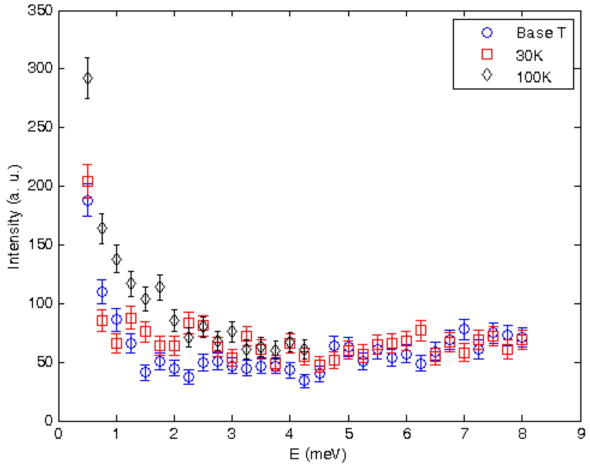

FIG. S7: **Inelastic measurement data on $CaRuO_3$**. Measurements were performed at T = 1.5 K (base temperature), 30 K and 100 K. Unlike $Ca(Co_{0.17}Ru_{0.83})O_3$, no excitation at finite energies are observed at any temperature in the stoichiometric compound (x = 0).

already divided by monitor counts. The standard Vanadium sample with 0.0373 mols was used on SPINS to find out N (number of unit cells) in $N_{vanadium}k_fR_0$. We performed incoherent inelastic scan using the Vanadium sample under identical instrument configuration used in our experiment.

In Figure S7, we show the inelastic measurement data on stoichiometric compound $CaRuO_3$ (x = 0) up to E = 8 meV of energy transfer. The measurements were performed at $Q \simeq 1$ $\AA^{-1}$. Unlike $Ca(Co_{0.17}Ru_{0.83})O_3$

compound where localized excitations are observed at finite energies (see Figure 6), no such inelastic behavior is observed in $CaRuO_3$.

[1] Department of Commerce and National Institute of Standards and Technology do not recommend any commercial product.
[2] N. W. Ashcroft, N. D. Mermin, *Solid State Physics*, Saunders College Publishing, 1976
[3] G. Xu, Z. Xu, J. M. Tranquada, *Rev. Sci. Inst.* 2013, **84**, 083906.
